# Structural Bases for the Fitness Cost of the Antibiotic-Resistance and Lethal Mutations at Position 1408 of 16S rRNA

**DOI:** 10.3390/molecules25010159

**Published:** 2019-12-31

**Authors:** Jiro Kondo, Mai Koganei

**Affiliations:** Department of Materials and Life Sciences, Faculty of Science and Technology, Sophia University, 7-1 Kioi-cho, Chiyoda-ku, Tokyo 102-8554, Japan; m.koganei511@gmail.com

**Keywords:** antibiotic resistance, fitness cost, decoding, molecular switch, ribosome

## Abstract

To understand a structural basis for the fitness cost of the A1408G antibiotic-resistance mutation in the ribosomal A-site RNA, we have determined crystal structures of its A1408C and A1408U lethal mutants, and made comparison with previously solved structures of the wild type and the antibiotic-resistant mutant. The A-site RNA containing an asymmetric internal loop functions as a molecular switch to discriminate a single cognate tRNA from several near-cognate tRNAs by its conformational ON/OFF switching. Overall structures of the “off” states of the A1408C/U lethal mutants are very similar to those of the wild type and the A1408G antibiotic-resistant mutant. However, significant differences are found in local base stacking interactions including the functionally important A1492 and A1493 residues. In the wild type and the A1408G antibiotic-resistant mutant “off” states, both adenines are exposed to the solvent region. On the other hand, one of the corresponding adenines of the lethal A1408C/U mutants stay deeply inside their A-site helices by forming a purine-pyrimidine AoC or A-U base pair and is sandwiched between the upper and lower bases. Therefore, the ON/OFF switching might unfavorably occur in the lethal mutants compared to the wild type and the A1408G antibiotic-resistant mutant. It is probable that bacteria manage to acquire antibiotic resistance without losing the function of the A-site molecular switch by mutating the position 1408 only from A to G, but not to pyrimidine base C or U.

## 1. Introduction

**Figure 1:**
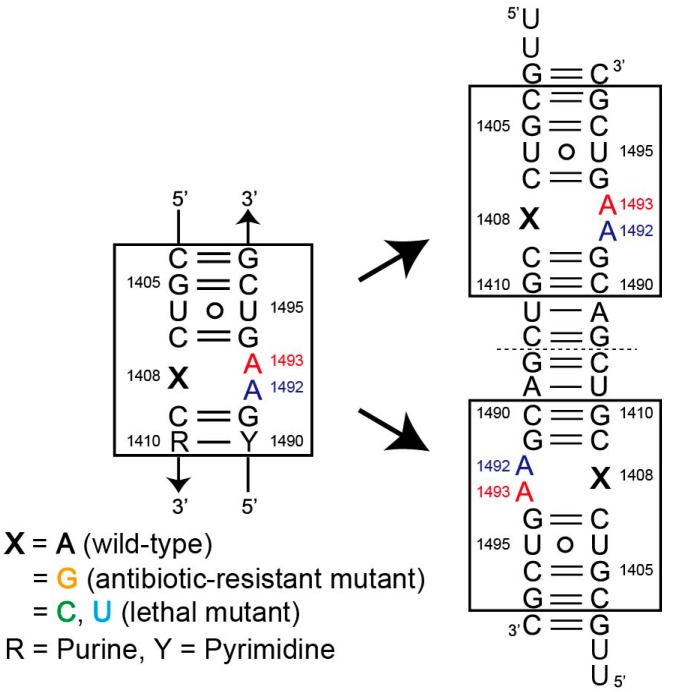
Secondary structures of the RNA duplexes used in our present and previous crystallographic studies. The two bacterial A-site molecular switches are boxed. A uracil residue at position 11 is replaced by a 5-bromouracil in bromine-derivatives. Two adenine residues A1492 and A1493 that recognize the codon-anticodon helix are colored in blue and red, respectively.

The aminoacyl-tRNA decoding site (A site) is one of the active sites of the ribosome where a single cognate tRNA is accurately discriminated from several near-cognate tRNAs [1,2,3,4]. In the decoding process, an RNA internal loop (Figure 1) composed of fifteen nucleotide residues functions as a molecular switch by changing its conformation between “off” and “on” states. Crystallographic studies of several ribosomal particles [5,6,7,8,9,10,11,12,13,14,15,16,17,18,19,20,21,22] as well as RNA fragments containing the A-site molecular switches [23,24,25,26,27,28,29,30] have revealed the molecular mechanism of the decoding process at atomic level, especially in the case of bacteria. Two consecutive adenines A1492 and A1493 in the asymmetric internal loop are the most important residues for the function of the molecular switch. In the absence of aminoacyl-tRNA, these two adenines can take various conformations, which are called as “off” states where both of or one of two adenines stay inside the A-site helix and form a base pair with the 1408 residue on the opposite strand. Once an aminoacyl-tRNA is delivered to and occupies the A site, the RNA molecular switch changes its conformation to a unique “on” state, in which two adenines A1492 and A1493 fully bulge out from the A-site helix, protrude toward the mRNA-tRNA complex and recognize the first two base pairs of the codon-anticodon mini-helix through A-minor motifs to check their geometries. If these base pairs are of the canonical Watson–Crick type, the aminoacyl-tRNA is recognized as the cognate one and can be accommodated by the ribosome. These two functional adenine residues are universally conserved as expected from their biological significances [31]. On the other hand, the 1408 residue on the opposite strand of the A-site internal loop must be a purine, but varies across the different organisms and organelles. For example, it is adenine in prokaryotes, mitochondria and chloroplasts, and is mostly guanine in eukaryotic cytoplasm [31].

A single chromosomal mutation at position 1408 of the bacterial 16S rRNA from A to G is the most prevalent antibiotic-resistant mutation found in clinical isolates [32,33,34,35,36,37,38,39,40,41]. The A1408G mutation confers high-level resistance against aminoglycosides with an ammonium group at position 6′ on ring I [42,43,44,45,46,47], and its structural basis has recently been revealed by our X-ray analyses; ring I with 6′-NH_3_^+^ can make a pseudo pair with A1408 of the wild type but cannot make the identical pseudo pair with G1408 of the antibiotic-resistant mutant due to repulsive force between 6′-NH_3_^+^ of ring I and N1-H and N2-H of G1408 [29]. In general, the antibiotic resistance is strongly associated with the fitness cost, which is observed as a reduced growth rate in the absence of antibiotics [48]. However, it was confirmed by Böttger and coworkers that the generation time is almost the same for the wild type (2.98 ± 0.06 h) and the A1408G antibiotic-resistant mutant (3.15 ± 0.06 h) strains of *Mycobacterium smegmatis* grown in broth culture [49], indicating that the A1408G antibiotic-resistant mutation does not affect the fitness of bacteria so much [49,50]. In our previous X-ray analyses, we have confirmed that the structures of the “off” and “on” states of the A1408G antibiotic-resistant mutant A site are very similar to those of the wild type A site. Therefore, it would appear that the A1408G antibiotic-resistant mutation does not disturb the function of the A-site molecular switch [29]. Now, an important question arises; why must the 1408 residue be a purine? Neither prokaryotic nor eukaryotic organisms have a pyrimidine at the position [31]. In fact, the A1408C and A1408U mutations are lethal to bacteria [49]. Another mutation experiment revealed that A1408U mutation significantly decreases protein expression [51]. Herein, we have performed X-ray analyses of RNA fragments containing the lethal-mutant A sites to see whether these mutations disturb the function of the RNA molecular switch.

## 2. Results and Discussion

### 2.1. Structures of the A1408C Lethal Mutant A Site and Its Comparison with the Wild Type and the A1408G Antibiotic-Resistant Mutant

**Figure 2:**
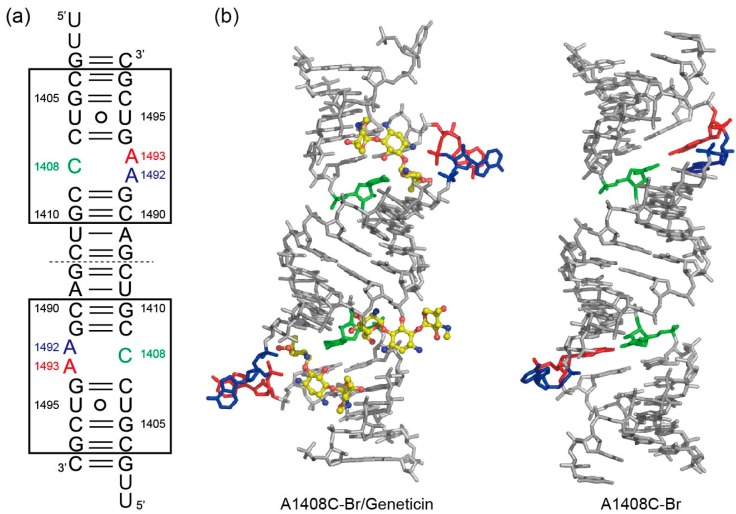
(**a**) Secondary structures of the RNA duplexes containing two A1408C mutant A-site molecular switches used in the crystallographic studies. (**b**) Tertiary structures of the RNA duplexes in the asymmetric unit of the A1408C-Br/geneticin and A1408C-Br crystals.

**Figure 3:**
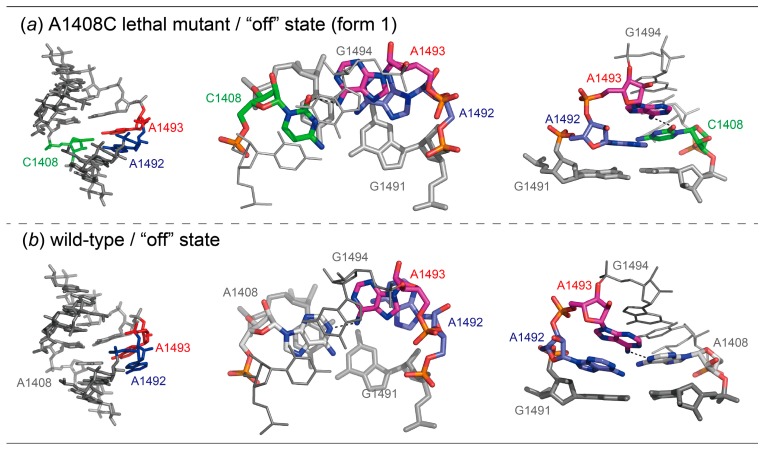
Molecular structures (left) and close-up views of base pairs (center) and stacking interactions (right) observed in the “off” state form 1 of the bacterial A1408C lethal mutant (**a**) and wild type (28: PDB-ID = 3BNL) (**b**) A sites.

**Figure 4:**
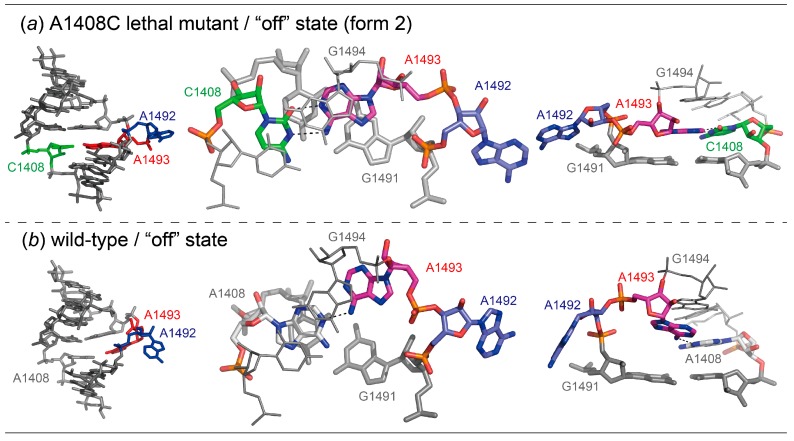
Molecular structures (left) and close-up views of base pairs (center) and stacking interactions (right) observed in the “off” state form 2 of the bacterial A1408C lethal mutant (**a**) and wild type (28: PDB-ID = 3BNL) (**b**) A sites.

**Figure 5:**
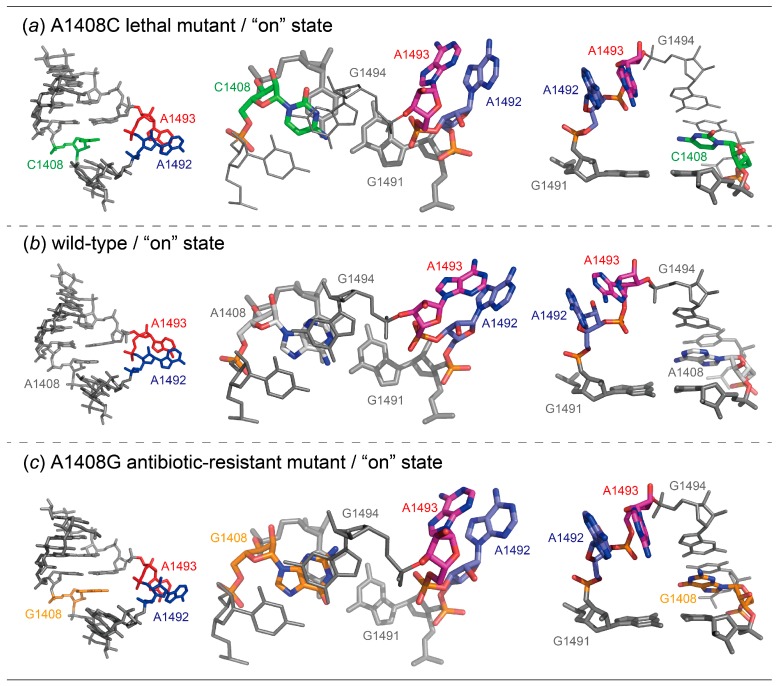
Molecular structures (left) and close-up views of base pairs (center) and stacking interactions (right) observed in the “on” state of the bacterial A1408C lethal mutant (**a**), wild type (12: PDB-ID = 2J00) (**b**) and the A1408G antibiotic-resistant mutant (29: PDB-ID = 3TD1) (**c**) A sites. For better understanding, geneticin and paromomycin bound to these A sites are removed.

For the A1408C lethal mutant A site, two types of crystals, A1408C-Br/geneticin and A1408C-Br, were obtained in the presence and absence of aminoglycoside geneticin, respectively (Figure 2). In each crystal, one RNA duplex is in the asymmetric unit, meaning that a total of four A-site switches are observed. In the absence of aminoglycoside, two A-site molecular switches take different “off” conformations (Figure 2b), one with bulged-in A1492 and A1493 (the “off” state form 1, hereafter; Figure 3a) and one with bulged-out A1492 and bulged-in A1493 (the “off” state form 2, hereafter; Figure 4a). In the presence of geneticin, the two A-site molecular switches are stabilized in the “on” state conformation with bulged-out adenines A1492 and A1493 (Figure 2b and Figure 5a).

In the “off” state form 1 (Figure 3a), the bulged-in A1493 forms a base pair with C1408 and stacks below G1494. The other bulged-in adenine A1492 does not make base pair in the A-site helix but stacks between A1493 and G1491. As a result, a stacking column G1494-A1493-A1492-G1491 is observed in the A-site helix. The “off” state form 1 is similar to the wild type “off” state with bulged-in A1492 and A1493 determined by our group (Figure 3 and Figure S1) [28]. In both cases, the bulged-in A1493 residue forms a base pair with the C/A1408 residue through only one hydrogen bond, N6_A1493_-H...O2_C1408_ or N6_A1493_-H...N1_A1408_. The C1′-C1′ distances are 10.8 Å for the AoC base pair and 12.5 Å for the AoA base pair, respectively. Therefore, the A1493 residue is inserted more deeply into the A-site helix in the A1408C lethal mutant than in the wild type. Difference is found also in the conformation of the other bulged-in adenine. In the A1408C lethal mutant, A1492 is deeply inserted into the A-site helix and sandwiched between A1493 and G1491, and is thereby involved in a long staking column from G1497 to C1490. In the wild type, however, the corresponding adenine residue A1492 is not inserted into the A-site helix, just stacks under A1493 and is partly exposed to the solvent region at the other side, so that two stacking columns from G1497 to A1492 and from G1491 to C1490 are formed. The distance between C1′ atoms of A1492 and C/A1408 are 12.5 and 14.5 Å, respectively. In consequence, the ribose rings of the A1492 and A1493 residues, which are fitted into the regular A-form helix in the A1408C lethal mutant “off” state form 1, are partly protruded from the A-site helix in the wild type “off” state.

In the “off” state form 2 (Figure 4a), the bulged-in A1493 forms a base pair with C1408. Since the neighboring A1492 residue fully bulges out from the A-site helix, the A1493 residue is intercalated between G1494 and G1491. The “off” state form 2 is similar to the wild type “off” state with bulged-out A1492 and bulged-in A1493 solved by our group (Figure 4 and Figure S2) [28]. In both A sites, A1492 bulges out from the A-site helix toward the solvent region where aminoacyl-tRNAs are delivered. In the A1408C lethal mutant, the bulged-in A1493 residue forms a base pair with C1408 through two hydrogen bonds N6_A1493_-H...N3_C1408_ and N1_A1493_-H...O2_C1408_. For making the base pair, the N1 atom of A1493 has to be protonated. In fact, the A^+^oC base pair is often observed in the 16S and 23S ribosomal RNAs [52,53], where RNA strands with acidic phosphate groups are compactly folded and packed. The C1′-C1′ distance of the A^+^oC base pair is 10.1 Å, which is slightly shorter than that of the canonical Watson–Crick base pairs (10.7 Å in average) [54]. The corresponding residue A1493 in the wild type “off” state forms a pair with A1408 through N6_A1493_-H...N1_A1408_ with the C1′-C1′ distance of 12.5 Å. Since A1493 of the A1408C lethal mutant is inserted more deeply into the A-site helix, the residue is intercalated between G1494 and G1491 and involved in a long stacking column. On the other hand, the A1493 residue of the wild type stacks only with G1494 and is partly exposed to the solvent region at the other side.

The “on” state of the A1408C lethal mutant is identical to that of the wild type as observed, for example, in the 70S ribosome structure solved by Ramakrishnan and coworkers [12], and also to that of the A1408G antibiotic-resistant mutant solved by our group [29] (Figure 5 and Figure S3). In the “on” state, both A1492 and A1493 largely bulge out from the A-site helix to recognize the mRNA-tRNA mini-helix, but make no intra-molecular interactions.

These observations suggest that the “off” state conformations are strongly correlated with the fact that the A1408C mutation is lethal to bacteria. The wild type A-site molecular switch can easily change its conformation from the “off” to “on” states, since the A1492 and A1493 residues essential for the decoding are partly exposed to the solvent region even in the “off” states. On the other hand, the corresponding adenine residues of the A1408C lethal mutant are stabilized in the long base-stacking column, and the ON/OFF switching might unfavorably occur in the lethal mutant compared to the wild type.

### 2.2. Structures of the A1408U Lethal Mutant A Site and Its Comparison with the Wild Type and the A1408G Antibiotic-Resistant Mutant

**Figure 6:**
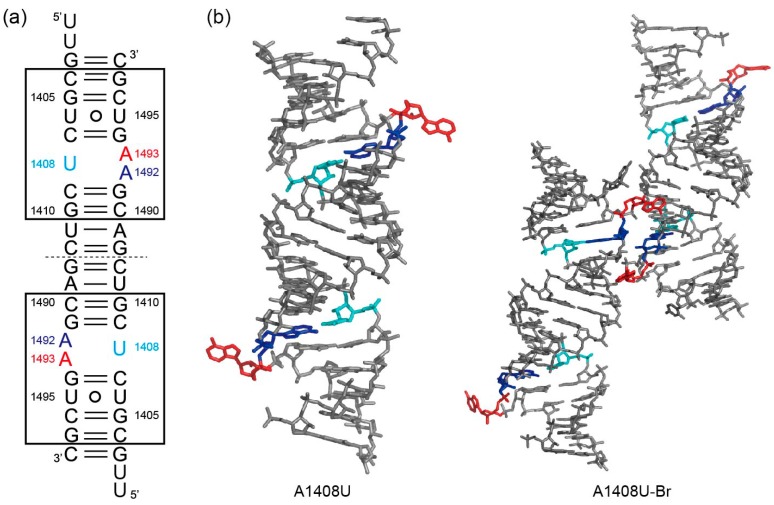
(**a**) Secondary structures of the RNA duplexes containing two A1408U mutant A-site molecular switches used in the crystallographic studies. (**b**) Tertiary structures of the RNA duplexes in the asymmetric unit of the A1408U/geneticin and A1408U-Br crystals.

**Figure 7:**
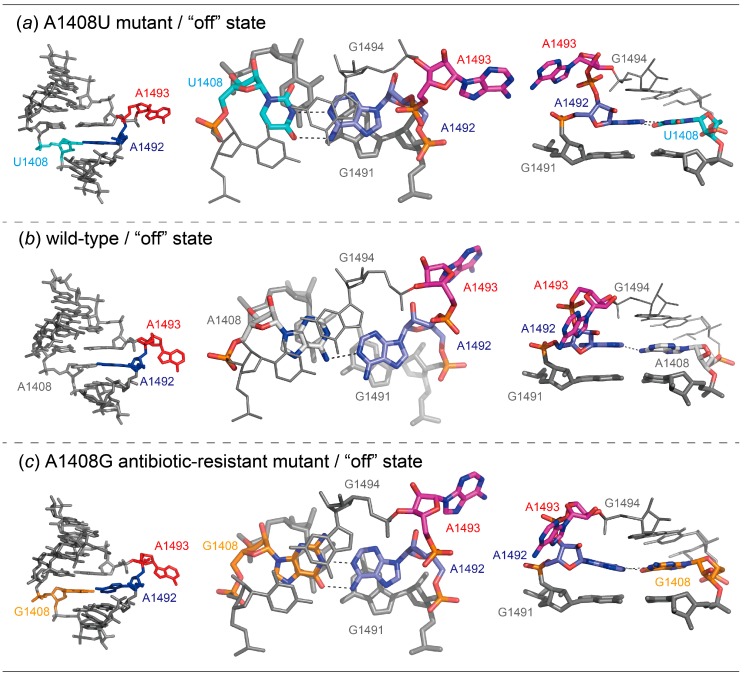
Molecular structures (left) and close-up views of base pairs (center) and stacking interactions (right) observed in the “off” state of the bacterial A1408U lethal mutant (**a**), wild type (24: PDB-ID = 1T0E) (**b**) and the A1408G antibiotic-resistant mutant (29: PDB-ID: 3TD0) (**c**) A sites.

For the A1408U lethal mutant A site, two different crystals, A1408U/geneticin and A1408U-Br, were obtained in the presence and absence of geneticin, respectively (Figure 6). In the A1408U/geneticin crystal, one RNA duplex is observed in the asymmetric unit, but electron density of geneticin is not observed. In the A1408U-Br crystal, two RNA duplexes are in the asymmetric unit. Therefore, a total of six A-site molecular switches are obtained from the two crystals. Interestingly, all these six A-site internal loops take an identical “off” state conformation with bulged-in A1492 and bulged-out A1493 (Figure 7a and Figure S4). The “off” state is similar to the wild type “off” state with bulged-in A1492 and bulged-out A1493 as solved by Hermann and coworkers [24] (Figure 7 and Figure S5). In addition, the “off” state is also similar to the A1408G antibiotic-resistant mutant “off” state determined by our group (Figure 7 and Figure S5) [29]. In these A sites, A1493 fully bulges out from the A-site helix and points toward the solvent region where aminoacyl-tRNAs are delivered. In the A1408U lethal mutant, the bulged-in A1492 residue forms a canonical Watson–Crick A-U base pair with U1408. The C1′-C1′ distance of the base pair is 11.1 Å. The corresponding residue A1492 in the wild type “off” state forms a pair with A1408 through N1_A1492_...H-N6_A1408_ with the C1′-C1′ distance of 12.3 Å. In the A1408G antibiotic-resistant mutant “off” state, a *cis* Watson–Crick A1492oG1408 base pair is formed through two hydrogen bonds N6_A1492_-H...O6_G1408_ and N1_A1492_…H-N1_G1408_ with the C1′-C1′ distance of 12.7 Å. Since the A1492 residue of the A1408U lethal mutant forms the canonical Watson–Crick A-U base pair, the residue is well fitted into the regular A-form helix and intercalated between G1494 and G1491. On the other hand, the corresponding residues of the wild type and the A1408G antibiotic-resistant mutant A sites stack only on G1491 and expose their base moieties to the solvent region at the other side.

From these observations, it would appear that the bulged-in A1492 residue of the wild type and the A1408G antibiotic-resistant mutant RNA molecular switches is likely to bulge out when the aminoacyl-tRNA is delivered to the A site. It is consistent with the fact that the generation time is almost the same for the wild type and the A1408G antibiotic-resistant mutant strains of *M. smegmatis* grown in broth culture [49]. However, the A1408U lethal mutant A site prefers to take a single stable “off” state where the A1492 residue is comfortably fitted into the A-site helix by forming the canonical Watson–Crick base pair with U1408 and stacking interactions with G1494 and G1491. It can be expected from the structural comparison that the ON/OFF switching of the A1408U lethal mutant A site might be energetically unfavorable compared to that of the wild type and the A1408G antibiotic-resistant mutant A sites.

### 2.3. Conclusions of Discussion

Bulges and internal loops are the most popular structural motifs observed in RNA molecules. However, little is known about their sequence-dependent structural and dynamic features. The ribosomal A-site internal loop composed of fifteen nucleotides including three highly-conserved residues, A/G1408, A1492 and A1493, has a characteristic feature that dynamically changes its conformation between the “off” (A1492 and/or A1493 are bulged in) and “on” (both A1492 and A1493 are fully bulged out) states, and functions as a molecular switch for discriminating a single cognate tRNA from several near-cognate tRNAs. In our previous studies [25,28,29], we have shown how small differences of nucleotide sequences affect the dynamics of the A-site molecular switches. Herein, we have further shown why the 1408 residue must be universally a purine. The 1408 residue strongly affects not only the base pair formation but also the stacking manner of A1492 and A1493 on the opposite strand. In the “off” state of the wild type and the A1408G antibiotic-resistant mutant A sites, the bulged-in A1492 or A1493 residue forming a base pair with A/G1408 are partly exposed to and are ready to bulge out toward the mRNA-tRNA helix for taking the “on” state. If the 1408 residue is a pyrimidine as observed in the A1408C/U lethal mutants, the A-site internal loop may lose its function as a molecular switch by taking the energetically stable “off” state where bulged-in A1492 or A1493 stays deeply inside the A-site helix by forming the purine-pyrimidine base pair, AoC or A-U. It is also important to note that the stability of the base pair between the residue at position 1408 and A1492/A1493 residues is not a critical factor, since the number of hydrogen bonds observed in the base pair is one or two in any case. It is probable that all organisms naturally select a purine at the position 1408, and bacteria can acquire antibiotic resistance without losing the function of the A-site molecular switch by mutating position 1408 from A to G, but not to pyrimidine base C or U.

## 3. Materials and Methods

### 3.1. Sample Preparations and Crystallizations

RNA duplexes containing two bacterial A-site molecular switches with a single A1408C or A1408U lethal point mutation were designed for the present crystallographic studies (Figure 1). Such RNA fragments have been extensively used as successful models in a series of crystallographic studies [23,24,25,26,27,28,29], since their crystal packing mimic highly-crowded environment of the A site in the ribosome. These RNA oligomers (A1408C and A1408U, hereafter), and their bromine derivatives for phase determinations (A1408C-Br and A1408U-Br, hereafter), were chemically synthesized by Dharmacon (Boulder, CO, USA) and GeneDesign (Osaka, Japan), and then purified by denaturing 20% polyacrylamide gel electrophoresis and C18 reversed-phase chromatography.

Crystallizations were performed in both the absence and presence of aminoglycoside geneticin (also known as G418). By analogy with our previous crystallographic studies of the bacterial wild type and the A1408G antibiotic-resistant mutant A sites [28,29], it can be expected that the lethal mutant A-site molecular switches may take the “off” state conformations in the absence of aminoglycoside. On the other hand, geneticin possessing strong affinities to both the wild type and the A1408G antibiotic-resistant mutant A sites [45] may have a potential to bind to and stabilize the “on” state of the lethal mutant switches in a similar way. Prior to crystallizations, 2 mM RNA solutions containing 100 mM sodium cacodylate (pH 7.0) were mixed with the same volume of 4 mM geneticin sulfate solution or distilled water. Crystallizations were performed by the hanging- and sitting-drop vapor diffusion methods at 293 K. Crystallization droplets were prepared by mixing 1 μL of RNA solutions and 1 μL of crystallization solutions containing 50 mM sodium cacodylate (pH 7.0), 1–10 mM spermine tetrahydrochloride, 10–300 mM monovalent or divalent cation chloride, 1%–10% (*v*/*v*) 2-methyl-2,4-pentanediol or polyethylene glycol 3350, and were equilibrated against 250 μL of reservoir solution containing 40% (*v*/*v*) 2-methyl-2,4-pentanediol or polyethylene glycol 3350. For the A1408C lethal mutant, rhomboid-shaped crystals and polycrystals were obtained in conditions with and without geneticin (crystal codes: A1408C-Br/geneticin, A1408C-Br), respectively (Table S1). A polycrystal of A1408C-Br was crashed with an ultrafine needle to obtain small pieces of single crystals. For the A1408U lethal mutant, cubic- and rod-shaped single crystals were obtained in conditions with and without geneticin (crystal codes: A1408U/geneticin, A1408U), respectively (Table S2). The A1408C-Br/geneticin, A1408C-Br and A1408U/geneticin crystals obtained in conditions containing 2-methyl-2,4-pentandiol were picked up with CryoLoops (Hampton Research, Aliso Viejo, CA, USA) and directly flash-cooled in liquid nitrogen before X-ray experiments. The A1408U crystal obtained in a condition containing polyethylene glycol 3350 was picked up with CryoLoop, transferred to a droplet of 40% 2-methyl-2,4-pentandiol (cryoprotectant), and then flash-cooled in liquid nitrogen.

### 3.2. Data Collections, Structure Determinations and Refinements

X-ray data of the four crystals were collected at 100 K with synchrotron radiation at the BL-5A and BL-17A beamlines of the Photon Factory (Tsukuba, Japan). For the phase determination with the multiple anomalous diffraction (MAD) method, the X-ray datasets of the A1408C-Br/geneticin and A1408C-Br crystals were taken with four different wavelengths. The datasets were processed and scaled with the program Crystalclear (Rigaku Americas, The Woodlands, TX, USA). The intensity data were further converted to structure-factor amplitudes using the program TRUNCATE from the CCP4 suite [55]. The statistics of data collections are summarized in Table S3.

Initial phases of the A1408C-Br/geneticin and A1408C-Br crystals were estimated by the MAD method using the program AutoSol from the Phenix suite [56,57,58] with figure-of-merit of 0.41 and 0.83, respectively. The molecular structures of A1408C-Br/geneticin and A1408C-Br were constructed with the program COOT [59,60]. Initial phases of the A1408U/geneticin and A1408U-Br crystals were determined by the molecular replacement method with the program AutoMR from the Phenix suite [56,61] using an originally constructed model of the A-form RNA duplex as a probe, and then construction of their molecular structures were completed by using the program COOT.

The atomic parameters of each structure were refined with the program CNS [62,63] through a combination of simulated-annealing, crystallographic conjugate gradient minimization refinements and B-factor refinements. The statistics of structure refinements are summarized in Table S1. Molecular drawings were made using the program PyMOL [64]. The atomic coordinates and experimental data of the A1408C-Br/geneticin, A1408C-Br, A1408U/geneticin and A1408U crystals have been deposited in the Protein Data Bank (PDB) with the ID codes 4P3S, 4P3T, 4P43 and 4P3U, respectively.

## Supplementary Materials

The following are available online, Supplementary Materials containing Tables S1–S3 and Figures S1–S5.
