# Supplementary material for: Structural Bases for the Fitness Cost of the Antibiotic-Resistance and Lethal Mutations at Position 1408 of 16S rRNA
Source: Molecules. 2019 Dec 31;25(1):159. doi: 10.3390/molecules25010159 (PMC6983231; doi:10.3390/molecules25010159)
Supplement: Supplementary file 1 [file molecules-25-00159-s001.pdf]

Supplementary Materials

for

Structural Bases for the Fitness Cost of the Antibiotic-Resistance and Lethal Mutations at Position 1408 of 16S rRNA

Jiro Kondo* and Mai Koganei

Department of Materials and Life Sciences, Faculty of Science and Technology, Sophia University, 7-1 Kioi-cho, Chiyoda-ku, 102-8554 Tokyo, Japan.

* Correspondence: j.kondo@sophia.ac.jp

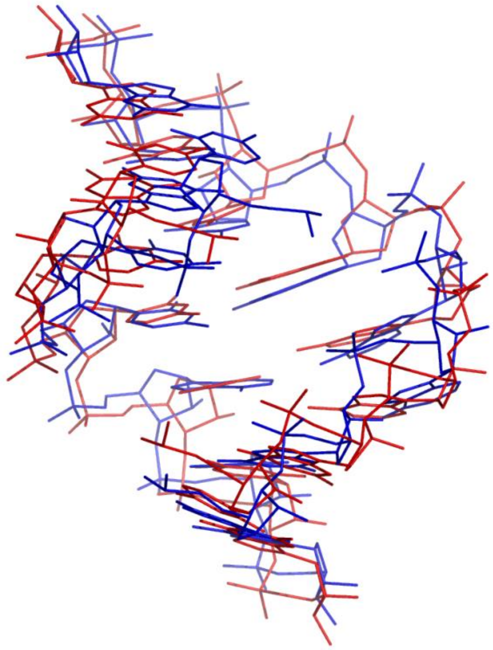

Figure S1. Superimposition between the "off" states form 1 of the A1408C mutant (red) and the wild type (blue: PDB-ID = 3BNL). The RMSD between them, excluding the C/A1408, G/A1410 and C/U1490 residues, is 1.4 Å.

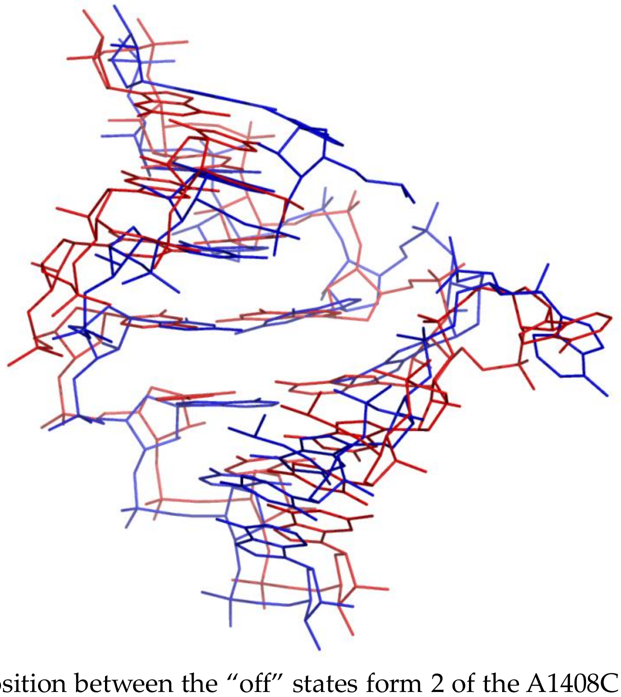

Figure S2. Superimposition between the "off" states form 2 of the A1408C mutant (red) and the wild type (blue: PDB-ID = 3BNL). The RMSD between them, excluding the C/A1408, G/A1410 and C/U1490 residues, is 2.2 Å.

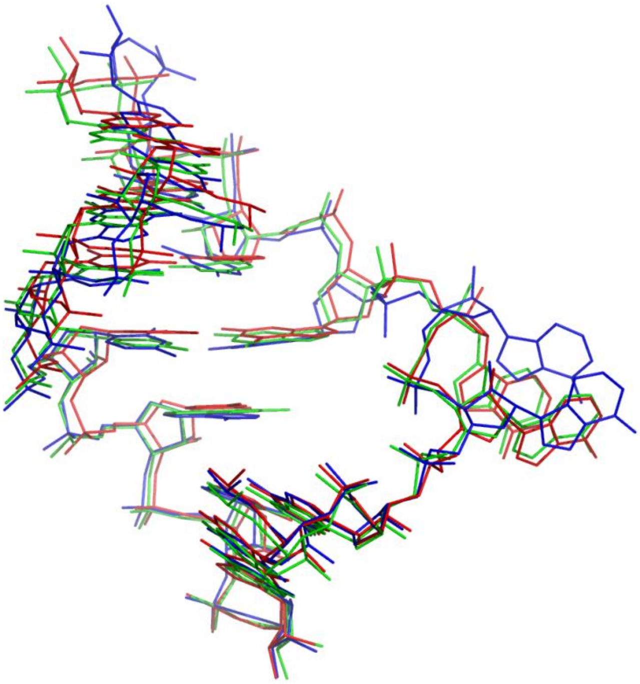

Figure S3. Superimposition between the "on" states of the A1408C mutant (red), the wild type (blue: PDB-ID = 2J00) and the A1408G antibiotic-resistant mutant (green: PDB-ID = 3TD1). The RMSDs among them, excluding the C/A/G1408, G/A1410 and C/U1490 residues, are in the range of 0.3-1.2 Å.

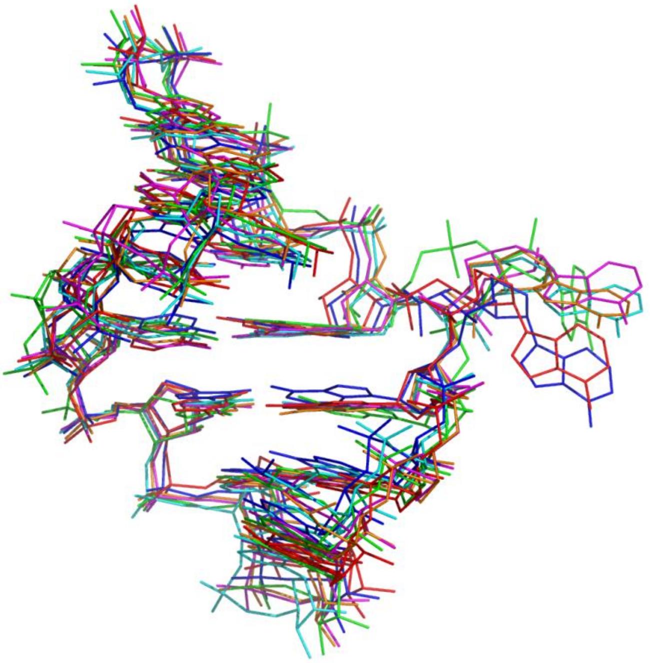

Figure S4. Superimpositions among the six independent copies (red, green, blue, magenta, cyan, orange) of the bacterial A1408U mutant A site obtained in the present study. The RMSDs among them are in the range of 0.8-1.8 Å.

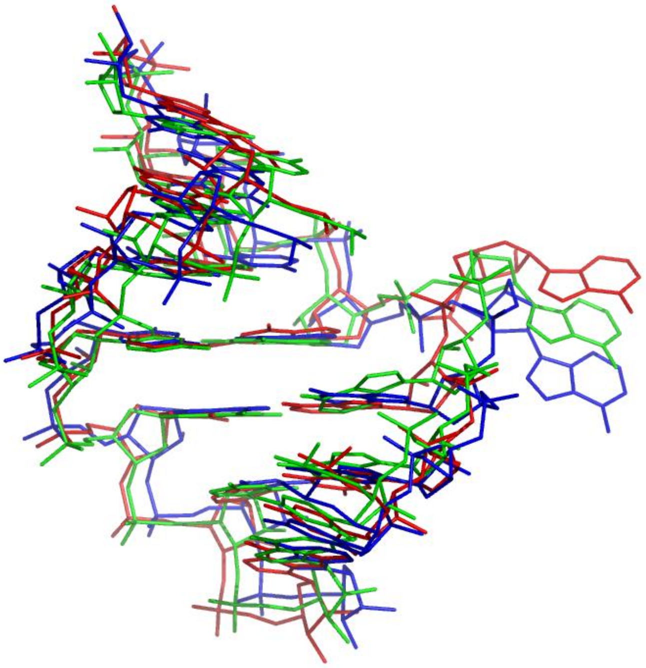

Figure S5. Superimposition between the "off" states of the A1408U mutant (red), the wild type (blue: PDB-ID = 1T0E) and the A1408G antibiotic-resistant mutant (green: PDB-ID = 3TD0). The RMSDs among them, excluding the U/A/G1408, G/A1410 and C/U1490 residues, are in the range of 1.2-1.7 Å.

Table S1. Crystallization conditions of the bacterial A1408C mutant A site.

Crystal code	A1408C-Br/geneticin	A1408C-Br
Temperature	293K	293K
<u>RNA solution (1 µl)</u>		
RNA (A1408C-Br)	1 mM	1 mM
Geneticin sulfate	2 mM	-
Sodium cacodylate (pH = 7.0)	50 mM	50 mM
<u>Crystallization solution (1µl)</u>		
Sodium cacodylate (pH = 7.0)	50 mM	50 mM
Spermine tetrahydrochloride	1 mM	10 mM
Ammonium chloride	-	300 mM
Sodium chloride	10 mM	-
2-Methyl-2,4-pentanediol	1%	10%
<u>Reservoir solution (250 µl)</u>		
2-Methyl-2,4-pentanediol	40%	40%
Crystals	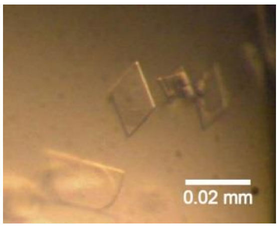	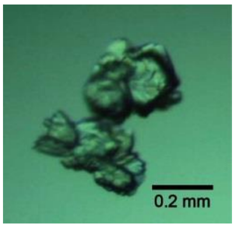

Table S2. Crystallization conditions of the bacterial A1408U mutant A site.

Crystal code	A1408U/geneticin	A1408U-Br
Temperature	293K	293K
<u>DNA solution (1 µl)</u>		
RNA (A1408U or A1408U-Br)	1 mM	1 mM
Geneticin sulfate	2 mM	-
Sodium cacodylate (pH = 7.0)	50 mM	50 mM
<u>Crystallization solution (1µl)</u>		
Sodium cacodylate (pH = 7.0)	50 mM	50 mM
Spermine tetrahydrochloride	1 mM	10 mM
Calcium chloride	10 mM	-
Sodium chloride		300 mM
2-Methyl-2,4-pentanediol	1%	-
Polyethylene glycol 3350	-	10%
<u>Reservoir solution (250 µl)</u>		
2-Methyl-2,4-pentanediol	40%	
Polyethylene glycol 3350	-	40%
Crystals	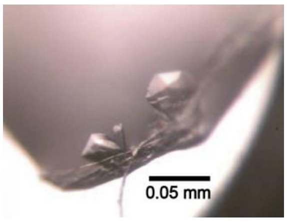	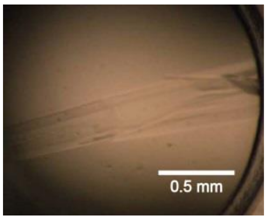

Table S3. Crystal data, statistics of data collections and structure refinements.

Crystal code	A1408C-Br/geneticin	A1408C-Br	A1408U/geneticin	A1408U-Br
PDB-ID	4P3S	4P3T	4P43	4P3U
<u>Crystal data</u>				
Space group	$P2_12_12_1$	$P2_1$	$C2$	$C2$
Unit cell (Å, °)	a = 34.1, b = 88.8, c = 46.6	a = 33.4, b = 40.5, c = 41.3<br>β = 103.1	a = 86.5, b = 31.7, c = 49.4<br>β = 116.3	a = 155.4, b = 32.7, c = 81.0<br>β = 117.1
Z [a]	1	1	1	2
<u>Data collection</u>				
Beamline	BL-5A of PF*	BL-5A of PF*	BL-17A of PF	BL-17A of PF
Wavelength (Å)	0.91938 / 0.920004 / 0.90659 / 1.00	0.91992 / 0.92005 / 0.90660 / 1.0	0.98	0.98
Resolution (Å)	44.3-2.3 / 44.4-2.3 / 44.4-2.3 / 44.4-2.3	32.7-1.7 / 32.7-1.7 / 32.6-1.7 / 40.2-1.6	44.3-2.0	25.6-3.0
of the outer shell (Å)	2.4-2.3 / 2.4-2.3 / 2.4-2.3 / 2.4-2.3	1.8-1.7 / 1.8-1.7 / 1.8-1.7 / 1.7-1.6	2.1-2.0	3.1-3.0
Unique reflections	12091 / 12108 / 12152 / 12128	23007 / 23062 / 23019 / 27284	8251	7533
Completeness (%)	99.8 / 99.8 / 99.9 / 99.7	98.6 / 98.7 / 98.8 / 98.1	99.2	99.4
in the outer shell (%)	99.9 / 99.9 / 100.0 / 100.0	100 / 100 / 100 / 99.9	97.5	100.0
R_{anom} [b] (%)	5.1 / 5.5 / 5.4/ 4 8	3.5 / 3.4 / 3.4 / 3.6	-	-
in the outer shell (%)	29.1 / 31.2 / 32.1 / 32.1	24.4 / 26.7 / 29.9 / 27.2	-	-
R_{merge} [c] (%)	-	-	7.2	9.1
in the outer shell (%)	-	-	37.1	37.7
Redundancy	3.7 / 3.7 / 3.7 / 3.7	3.8 / 3.8 / 3.8 / 3.7	3.6	3.5
in the outer shell	3.8 / 3.8 / 3.8 / 3.8	3.7 / 3.7 / 3.8 / 3.7	3.5	3.6
<u>Structure refinement</u>				
Resolution range (Å)	44.4-2.3	20.0-1.6	44.3-2.0	25.6-3.0
Used reflections	6694	14085	8250	7532
R-factor [d] (%)	23.0	24.8	24.6	29.5
R_{free} [e] (%)	27.0	27.1	27.0	30.2
Number of aminoglycosides	3	-	-	-
Number of ions	-	-	2 Ca^{2+}, 2 Na^{+}	-
Number of water	68	150	125	14
R.m.s.d. bond length (Å)	0.006	0.004	0.004	0.008
R.m.s.d. bond angles (°)	0.9	0.9	0.8	1.3

[a] Number of dsRNA in the asymmetric unit.

[b] $R_{anom} = 100 \times \Sigma_{hklj} | I_{hklj}(+) - I_{hklj}(-) | / \Sigma_{hklj}[I_{hklj}(+) + I_{hklj}(-)]$.

[c] $R_{merge} = 100 \times \Sigma_{hklj} | I_{hklj} - <I_{hklj}> | / \Sigma_{hklj}<I_{hklj}>$.

[d] R-factor = $100 \times \Sigma | |F_o| - |F_c| | / \Sigma |F_o|$, where $|F_o|$ and $|F_c|$ are optimally scaled observed and calculated structure factor amplitudes, respectively.

[e] Calculated using a random set containing 10% of observations.

* For phase determination with the multiple anomalous diffraction (MAD) method, four datasets were collected with four wavelengths. Statistics from left to right are of peak, edge and two remote data, respectively
